# Microstructure and Mechanical Property of 6082 Aluminum Alloy via Sc and Zr Addition Combined with Squeeze Casting

**DOI:** 10.3390/ma18091988

**Published:** 2025-04-27

**Authors:** Yushi Qi, Fangming Wei, Yu Wang, Yu Jin, Xusheng Chang, Gang Chen

**Affiliations:** School of Materials Science and Engineering, Harbin Institute of Technology, Weihai 264209, China; qys_gd@sina.cn (Y.Q.);

**Keywords:** 6082 aluminum alloy, squeeze casting, Scandium, Zirconium, mechanical properties

## Abstract

To enhance the mechanical properties of 6082 aluminum alloy, a novel Sc- and Zr-microalloyed 6082 alloy was fabricated through squeeze casting technology. Microalloying with Sc and Zr substantially refined the microstructure of alloy, achieving an average grain size of 136.36 μm—a 31.7% reduction compared to the baseline 6082 alloy. Furthermore, the addition of Sc and Zr effectively refined the coarse AlFeMnSi intermetallic phases, mitigating their inherent brittleness. The Sc/Zr-modified alloy exhibited delayed age-hardening kinetics, requiring 100% longer aging time to reach peak hardness due to Sc/Zr-induced retardation of β’’-phase precipitation. The optimized alloy demonstrated better mechanical properties, showing 10.4%, 8.0%, and 71.8% enhancements in yield strength, ultimate tensile strength, and elongation, respectively, over the non-microalloyed counterpart. The squeeze-cast Sc/Zr-modified alloy valve body showed yield strength exceeding 300 MPa and elongation above 10% across various sections, which verifies the effectiveness of this integrated microalloying and forming approach.

## 1. Introduction

The rapid growth of global automobile production has intensified energy demands and environmental pollution, driving urgent needs for vehicle lightweighting to enhance fuel efficiency and reduce carbon emissions. Consequently, aluminum alloys have become a key research focus for automotive body structures [1,2,3,4,5].

Current manufacturing approaches for complex aluminum alloy components present critical technical trade-offs. Conventional casting methods (gravity/low-pressure) enable intricate geometries but suffer from inherent defects including shrinkage porosity and incomplete mold filling [6]. Alternative processing routes combining plastic deformation (extrusion/forging) with machining achieve dimensional precision at the expense of material yield and mechanical integrity due to flow line discontinuity [7]. These limitations have accelerated development of aluminum near-net-shaping technologies that optimize performance–cost ratios through minimized machining and enhanced microstructural control [8].

Squeeze casting technology synergistically combines casting’s shape forming capability with forging’s microstructure control, establishing itself as a premier near-net-shape manufacturing solution for high-performance aluminum components [9]. The process applies controlled high pressure to molten metal during solidification, achieving dual benefits: eliminating casting defects through enhanced melt feeding, and inducing plastic deformation that generates refined equiaxed grains and optimized dislocation networks. This unique solidification–deformation coupling mechanism produces components possessing better strength-to-weight ratios compared to conventional castings while achieving high dimensional accuracy relative to machined counterparts [10,11,12].

While squeeze casting provides substantial advantages, its standalone application remains insufficient to satisfy escalating market demand for high-performance aluminum alloy components. To further optimize the mechanical properties and microstructure, researchers have actively explored microalloying techniques that synergistically modify both mechanical properties and microstructural characteristics. Strategic incorporation of selected rare earth elements (REs), including Sc, Ce, La, and Er, has become an effective approach for performance enhancement [13,14,15,16]. These elements effectively inhibit recrystallization, refine grain structure, and promote the formation of nano-scale Al_3_M (M = Sc, Er, Y, etc.) precipitates that strengthen the matrix. Given the economic constraints associated with rare earth utilization, researchers focus on hybrid microalloying systems combining REs with transition metals (TMs, such as Zr, Cr, Ti) [17,18,19,20]. This strategy facilitates the development of core-shell nanostructured precipitates that optimize cost–performance ratios. A study by Leng et al. [21] on 7075 aluminum alloy reveals that the combined addition of Sc and Zr generates coherent Al_3_(Sc, Zr) precipitates, producing simultaneous 21.7% tensile strength enhancement and 25.0% elongation improvement through dislocation pinning and grain boundary stabilization. Complementary research by Deng et al. [22] on Al-Zn-Mg-Sc-Zr alloys demonstrated 96 MPa and 58 MPa increments in yield and ultimate tensile strengths, respectively, while maintaining more than 12% elongation. These improvements primarily originate from Orowan strengthening mechanisms introduced by Al_3_(Sc, Zr) precipitates formed via Sc and Zr microalloying in the aged Al-Zn-Mg alloy.

As a representative Al-Mg-Si series alloy, 6082 aluminum has gained extensive application in automotive structural components due to its balanced strength–formability-–corrosion triad [23,24,25]. However, conventional heat treatments typically induce an inherent strength–ductility trade-off, where strength enhancement accompanies ductility reduction [26]. Recent advances suggest this limitation can be overcome through strategic microalloying with selected rare earth elements additions.

In this study, a Sc/Zr-modified 6082 aluminum alloy containing 0.3 wt.% Scandium (Sc) and 0.2 wt.% Zirconium (Zr) was successfully fabricated via squeeze casting method. The Sc/Zr-induced microstructural evolution and resultant mechanical property enhancement mechanisms were systematically examined. Age-hardening kinetics were quantitatively characterized through isothermal aging at 180 °C (0–16 h) with Vickers hardness measurements. Building upon these insights, asymmetric 6082 aluminum alloy components were successfully fabricated via squeeze casting, achieving concurrent strength improvement and ductility preservation.

## 2. Materials and Methods

### 2.1. Materials Fabrication

In this study, commercial 6082 aluminum alloy rods along with Al-2Sc and Al-10Zr master alloys were used as raw materials. All materials were obtained from Xinyue Chemical and Glass Co., Ltd. (Weihai, Shandong, China). The squeeze casting technique was used to fabricate the baseline 6082 alloy and a modified version containing 0.3 wt.% Sc and 0.2 wt.% Zr (designated as Sc-Zr-6082 alloy).

The preparation procedure involved the following steps. Approximately 800 g of 6082 aluminum alloy was weighed and loaded into a graphite crucible preheated to 300 °C, followed by complete melting at 700 °C in a resistance furnace. Upon complete melting, pre-calculated amounts of Al-2Sc alloy (18.1 wt.% of 6082 alloy) and Al-10Zr alloy (2.5 wt.% of 6082 alloy) were introduced into the melt to attain the target Sc/Zr concentrations. The molten alloy was then mechanically stirred at 300 rpm for 15 min to ensure homogeneity. Degassing treatment was subsequently conducted by adding 9 g of hexachloroethane (C_2_Cl_6_) wrapped in aluminum foil (1.0 wt.% of melt mass), followed by a 5 min settling period for slag removal.

**Figure 1:**
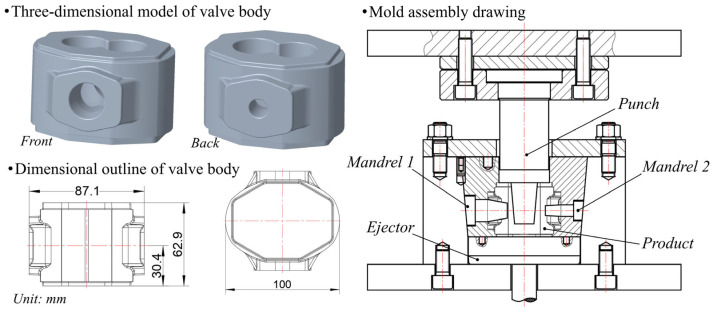
Valve body three-dimensional model, dimensional outline, and mold assembly drawing.

The molten alloy was poured into two distinct mold configurations according to experimental needs. For preliminary testing, a cylindrical steel mold (50 mm inner diameter × 120 mm height) coated with a graphite lubricant was employed. The valve body three-dimensional model, dimensional outline, and mold assembly drawing are shown in Figure 1 (the specific parameters of the mold can be referenced from previous related studies [8]). Both molds were uniformly preheated to 300 °C, with squeeze casting performed using a 1000 kN servo-hydraulic press. The casting parameters included an applied pressure of 150 MPa, loading velocity of 5 mm/s, and pressure dwell time of 30 s to ensure complete solidification.

To systematically investigate the influence of aging time on the mechanical properties of Sc/Zr-modified alloys, the aging temperature (180 °C) was selected following established protocols derived from prior studies [24,27,28]. The samples were subjected to solution treatment at 530 °C for 10 h with water quenching, followed by time-dependent aging treatments at the designated temperature.

The chemical composition of squeeze-cast alloys was quantitatively determined using inductively coupled plasma mass spectrometry (ICP-MS), with detailed chemical analysis results shown in Table 1.

### 2.2. Material Characterizations

Microstructural characterization and elemental analysis were conducted using an Olympus optical microscope (OM, Tokyo, Japan) and a Zeiss Merlin scanning electron microscope (SEM, Oberkochen, Germany) equipped with energy-dispersive X-ray spectroscopy (EDS). Specimens for OM examination were mechanically ground using SiC abrasive papers (400–2000 grit, Xinyue Chemical and Glass Co., Ltd., Weihai, Shandong, China) followed by sequential polishing with 2.5 μm and 0.5 μm diamond suspensions. Keller’s reagent (95 vol.% H_2_O, 2.5 vol.% HNO_3_, 1.5 vol.% HCl, 1 vol.% HF, Aladdin Chemical Co., Ltd., Shanghai, China) was employed for etching OM samples for 10 s. Transmission electron microscopy (TEM) and high-resolution TEM (HRTEM) analyses were carried out on a FEI Talos F200X TEM (Thermo Fisher Scientific, Hampton, N.H., USA) operated at 200 kV. High-angle annular dark-field scanning transmission electron microscopy (HAADF-STEM) imaging was performed with an aberration-corrected FEI Titan Cubed Themis G2 TEM (Thermo Fisher Scientific, Hampton, N.H., USA) at 300 kV. All TEM observations were conducted along the <110> zone axis of aluminum. The samples were initially mechanically thinned to a thickness of 30–50 μm, followed by argon ion milling. Grain size distribution was statistically quantified via electron backscatter diffraction (EBSD) analysis. Prior to EBSD, samples were electropolished in a solution of 90% ethanol and 10% perchloric acid at 15 V for 60 s.

### 2.3. Mechanical Testing

Vickers hardness testing was measured using a Future-Tech FM-700 tester (Tokyo, Japan) on cubic samples (10 × 10 × 10 mm^3^) with a 200 gf load and 15 s dwell time. Eight measurements per sample ensured statistical reliability. The tensile tests were conducted on an Instron-5967 universal testing machine at a constant strain rate of 0.001 s^−1^. Triplicate tests provided averaged mechanical properties. The elongation rate was calculated as (*b* − *a*)/*a* × 100%, where *a* and *b* represent initial and final gauge lengths, respectively [27].

## 3. Results and Discussion

### 3.1. As-Cast Microstructure and Properties of 6082 Alloy and Sc-Zr-6082

**Figure 2:**
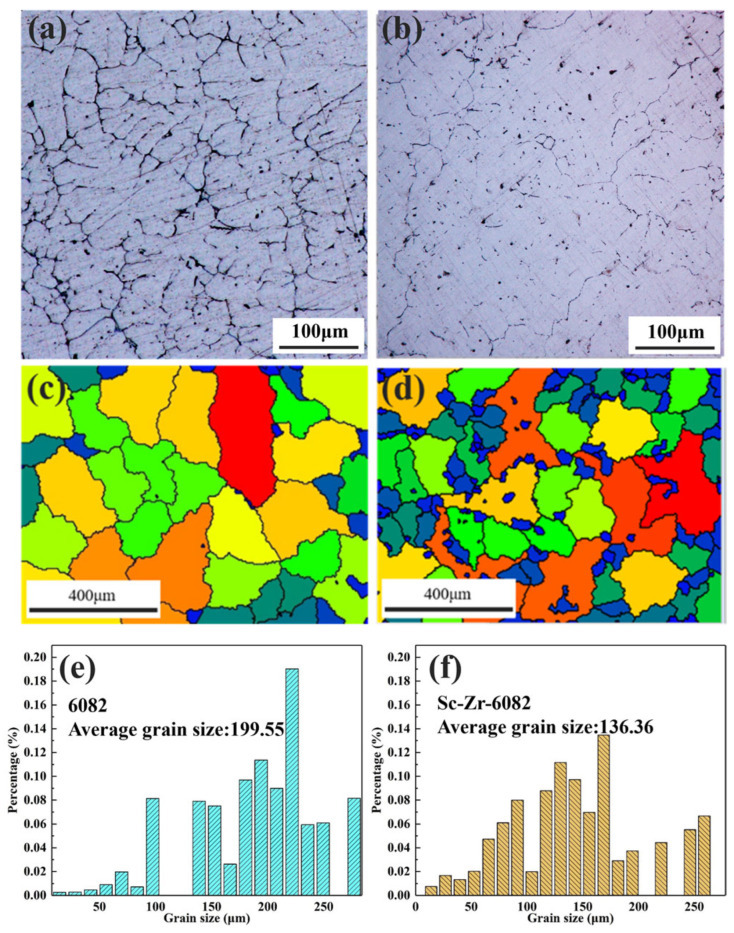
As-cast metallographic images and grain size distribution of 6082 alloy and Sc-Zr-6082 alloy: (**a**,**c**,**e**) for 6082 alloy; (**b**,**d**,**f**) for Sc-Zr-6082 alloy.

Figure 2 compares the metallographic microstructure and grain size distribution of the squeeze-cast 6082 alloy and Sc-Zr-6082 alloy, emphasizing the microstructural evolution induced by Sc/Zr microalloying. As shown in Figure 2a, the 6082 alloy exhibits characteristic dendritic structure with an average secondary dendrite arm spacing (SDAS) of 27.19 μm. Conversely, as shown in Figure 2b, the Sc-Zr-6082 alloy demonstrates substantial microstructural refinement, transitioning from dendritic to equiaxed grain morphology. Quantitative EBSD analysis (Figure 2e,f) further validate this transformation, revealing respective average grain sizes of 199.55 μm (6082 alloy) and 136.36 μm (Sc-Zr-6082 alloy), representing a 31.7% reduction. These results demonstrate that the Sc/Zr co-addition effectively promotes grain refinement in 6082 alloy during squeeze casting.

The primary mechanism of grain refinement by rare earth elements lies in their extremely limited solid solubility in α-Al matrix. During the solidification process, rare earth elements segregate at the solid–liquid interface, impeding the growth of α-Al grains, thereby achieving grain refinement. Additionally, this interfacial segregation induces constitutional undercooling, which promotes dendritic branching and reduces dendrite arm spacing, further contributing to microstructural refinement [2,29,30].

**Figure 3:**
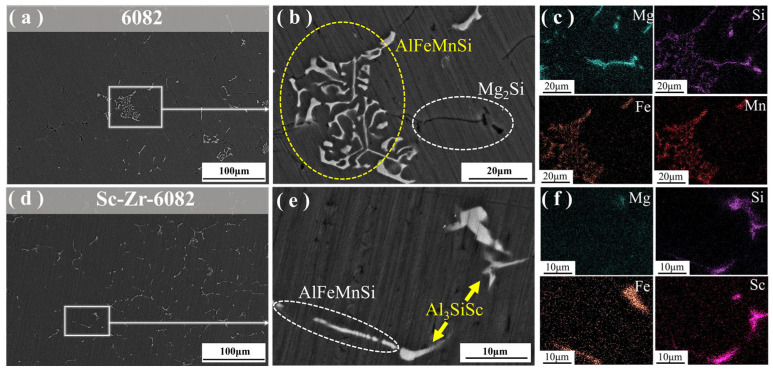
SEM images of 6082 alloy and Sc-Zr-6082 alloy: (**a**–**c**) for 6082 alloy; (**d**–**f**) for Sc-Zr-6082 alloy.

Figure 3 presents the SEM images of 6082 alloy and Sc-Zr-6082 alloy. As shown in Figure 3a,d, the 6082 alloy exhibits substantially coarser grains compared to the Sc-Zr-6082 alloy. The intermetallic phases in the 6082 alloy also demonstrate larger morphological dimensions. EDS analysis was conducted on the predominant intermetallic phases, with the results shown in Figure 3c. In Figure 3b, the 6082 alloy contains black rod-like and white skeletal secondary phases. The EDS mapping results indicate that the matrix is primarily composed of Al, with minor amounts of Si, Mn, and Fe, suggesting that the matrix is an α-Al solid solution. The white skeletal phase was identified as the AlFeMnSi intermetallic phase. Due to the similar physical and chemical properties of Fe and Mn, regions rich in Fe often exhibit Mn segregation. This indicates that Mn may substitute for Fe during the formation of the crystalline phase, resulting in the AlFeMnSi quaternary phase. These coarse AlFeMnSi intermetallic compounds exhibit inherent brittleness that may adversely affect the alloy’s performance. Figure 3c indicates that the black rod-like phase is Mg_2_Si. Notably, as shown in Figure 3e, the addition of Sc and Zr significantly alters the morphology of these compounds. Two new intermetallic compounds were observed at the grain boundaries and identified through elemental distribution analysis as AlScSi and AlFeMnSi intermetallic compounds. Sc combines with aluminum and silicon to form the AlScSi phase, competing with the pre-existing AlFeSi phase for grain boundary sites. Additionally, with the introduction of Sc and Zr, the morphology of the AlFeMnSi phase changes from skeletal to rod-like, configurations with marked dimensional refinement. This microstructural evolution is crucial for improving the alloy’s performance.

The 6082 alloy contains primary constituents Al-Mg-Si with minor additions of Mn and Fe. During solidification, these alloying elements tend to segregate at the solid–liquid interface, leading to the formation of coarse, hard, brittle, and highly thermally shrinkable AlFeMnSi phases [31], as shown in Figure 3b. These detrimental phases are typically regarded as impurity phases in Al-Mg-Si alloys, causing significant fragmentation in the matrix, which results in increased hot brittleness and a higher tendency for crack formation in the ingot. Additionally, Fe-rich phases can act as stress concentration sites, reducing both the strength and ductility of the alloy. The addition of 0.3 wt.% Sc and 0.2 wt.% Zr induces morphological modification of the Fe-rich phases, transitioning from needle-like or coarse skeletal shapes to fine, fragmented, spheroidal structures with rounded edges. This microstructural refinement effectively mitigates the negative effects of the Fe-rich impurity phases, preventing stress concentration during tensile testing and consequently improving the strength and ductility of the alloy.

**Figure 4:**
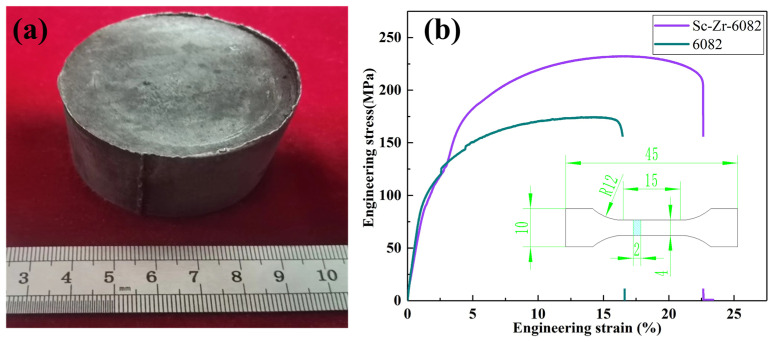
Squeeze-cast ingot and room-temperature tensile test results of 6082 alloy and Sc-Zr-6080 alloy: (**a**) squeeze-cast ingot, (**b**) stress–strain curve.

Figure 4a presents the squeeze-cast ingot of the 6082 alloy and the Sc-Zr-6082 alloy. Tensile specimens were extracted from the central portions of both ingots to ensure consistent mechanical property evaluation. Standardized tensile testing confirmed the exceptional room-temperature mechanical performance of the Sc-Zr-6082 alloy compared to the baseline 6082 alloy, as shown in Figure 4b.

A comparative evaluation of room-temperature mechanical properties between the squeeze-cast 6082 alloy and Sc-Zr-6082 alloy reveals an interesting phenomenon: the addition of Sc and Zr not only increased the alloy’s strength but also improved its ductility. This enhancement contrasts with traditional microalloying methods, which typically increase strength at the expense of elongation. As shown in Table 2, the 6082 alloy exhibits yield strength (YS) of 93.1 MPa, ultimate tensile strength (UTS) of 171.3 MPa, and elongation (EL) of 19.2%. The Sc-Zr-6082 alloy exhibits significantly improved mechanical performance with YS of 123.4 MPa, UTS of 238.9 MPa, and EL of 22.8%, respectively, representing enhancements of 32.5%, 39.5%, and 18.8% compared to the 6082 alloy.

**Figure 5:**
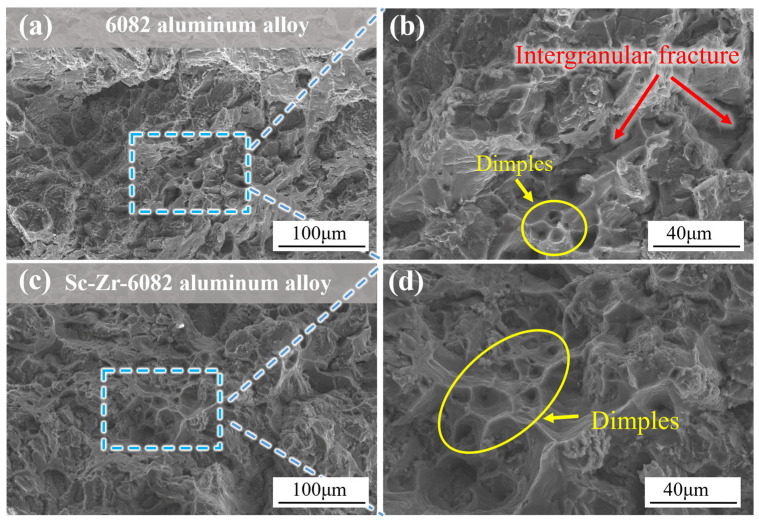
SEM fracture micrographs of tensile samples of 6082 alloy and Sc-Zr-6082 alloy: (**a**,**b**) the 6082 alloy; (**c**,**d**) the Sc-Zr-6082 alloy.

Figure 5 display the SEM fracture surfaces of the squeeze-cast 6082 alloy and the Sc-Zr-6082 alloy at low and high magnifications. The Sc-Zr-modified alloy exhibits a higher density of finer and deeper dimples compared to the 6082 alloy. As shown in Figure 5a,b, the unmodified 6082 alloy displays a mixed fracture mode characterized by pits and cleavage planes. In contrast, the Sc-Zr-6082 alloy shows a notable increase in dimples and an almost complete absence of brittle fracture marks due to the grain refinement effect, as shown in Figure 5c,d, demonstrating a clear ductile fracture mode.

### 3.2. Mechanical Properties of the Alloy After Heat Treatment

**Figure 6:**
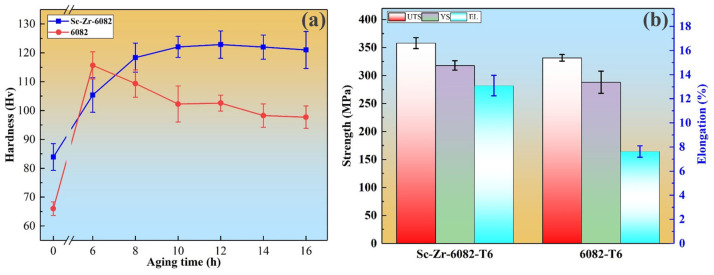
Hardness and mechanical properties at peak-aged state of 6082 alloy and Sc-Zr-6082 alloy: (**a**) hardness variation with time during aging at 180 °C; (**b**) mechanical properties at peak-aged state.

Figure 6a illustrates the Vickers hardness evolution of the squeeze-cast 6082 alloy and Sc-Zr-6082 alloy during isothermal aging at 180 °C. The “0 h” datum represents the as-cast condition prior to heat treatment. It is evident from Figure 6 that the addition of Sc and Zr significantly enhances the initial hardness of the alloy, indicating that these elements play a positive role in strengthening the alloy. As the aging time increases, the hardness rises rapidly, with the 6082 alloy reaching peak hardness after 6 h, at which point its hardness exceeds that of the Sc-Zr-6082 alloy. However, with further aging, the hardness of the 6082 alloy gradually decreases. In contrast, the Sc-Zr-6082 alloy exhibits a more stable hardness increase during the initial aging phase, reaching its peak hardness at 12 h. Even with further aging, the hardness of the Sc-Zr-6082 alloy decreases only slightly, demonstrating better aging stability. This result aligns with the findings reported in reference [32].

To investigate the mechanical properties in the peak-aged condition, tensile tests were conducted at room temperature after specified aging treatments. As shown in Figure 6b, the yield strength (YS), ultimate tensile strength (UTS), and elongation (EL) of the 6082 alloy were 288.3 MPa, 331.6 MPa, and 7.6%, respectively. In contrast, after T6 heat treatment, the YS, UTS, and EL of the Sc-Zr-6082 alloy reached 318.5 MPa, 358.0 MPa, and 13.1%, respectively, representing increases of 10.4%, 8.0%, and 71.8% compared to the unmodified alloy.

**Figure 7:**
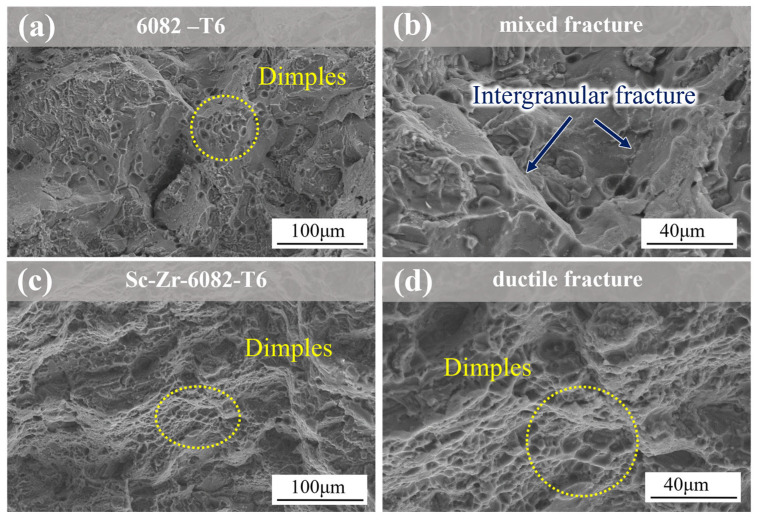
SEM fracture micrographs of tensile samples of 6082 alloy and Sc-Zr-6082 alloy after heat treatment: (**a**,**b**) the 6082 alloy; (**c**,**d**) the Sc-Zr-6082 alloy.

Figure 7 presents the SEM fracture surfaces of 6082 alloy and Sc-Zr-6082 alloy after T6 heat treatment at low and high magnifications. Compared to the as-cast alloys, the heat-treated alloys exhibit reduced cleavage features and increased dimple density, attributed to the spheroidization of eutectic silicon during the heat treatment process. As shown in Figure 7a,b, the unmodified alloy retains a mixed fracture mode. However, with the addition of Sc and Zr, the fracture surface reveals an abundance of smaller and deeper dimples, as depicted in Figure 7c,d. This indicates a greater contribution of ductile fracture mechanisms upon the addition of Sc and Zr. These observations are consistent with the mechanical properties obtained from the tensile tests in this study.

**Figure 8:**
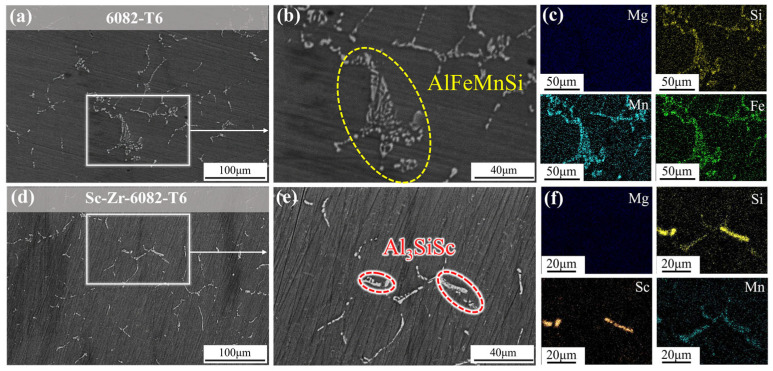
SEM images of 6082 alloy and Sc-Zr-6082 alloy after heat treatment: (**a**–**c**) 6082 alloy; (**d**–**f**) Sc-Zr-6082 alloy.

Figure 8 presents SEM images of the 6082 alloy and Sc-Zr-6082 alloy after heat treatment. The images show discontinuous features at the grain boundaries of both alloys. This discontinuity is primarily attributed to the dissolution of intermetallic compounds during the solution treatment, followed homogeneous precipitation of secondary phases during subsequent aging. Notably, Figure 8d,f reveal significant grain refinement in the squeeze-cast Sc-Zr-6082 alloy. This grain refinement is primarily due to the synergistic interaction between intermetallic compounds and the applied pressure applied during the squeeze casting process, effectively suppressing grain growth.

**Figure 9:**
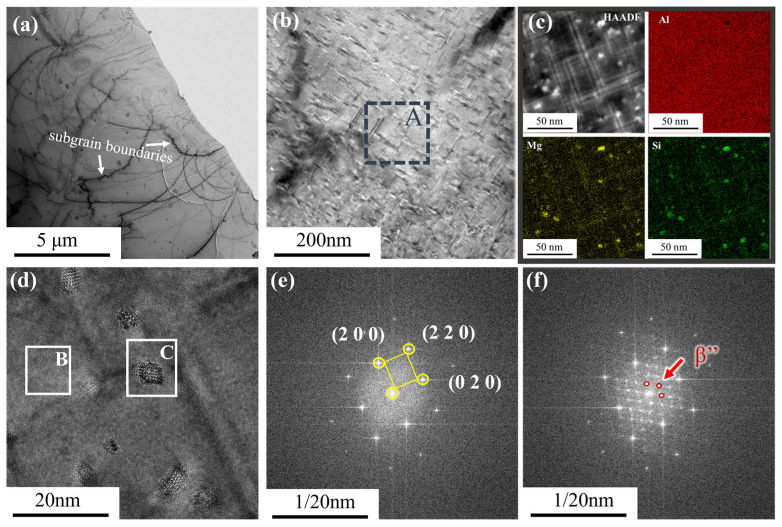
TEM characterization of the Sc-Zr-6082 alloy after heat treatment: (**a**,**b**) bright field images, (**c**) HAADF image of region A, (**d**) HRTEM image, (**e**) FFT of region B, (**f**) FFT of region C.

Figure 9 presents TEM images of the Sc-Zr-6082 alloy in the peak-aged state, observed along the <110> crystal zone axis. Figure 9a shows a significant accumulation of dislocation tangles along the sub-grain boundaries, formed by dislocation cross-slip during plastic deformation. Figure 9b illustrates the coexistence of two distinct precipitate morphologies under peak-aging conditions: needle-shaped precipitates (<10 nm) and rod-shaped precipitates (40–80 nm). Notably, the number density of the needle-like precipitates is significantly higher than that of the rod-like precipitates, suggesting preferential formation of the needle-like precipitates under the applied aging parameters. Elemental mapping in Figure 9c shows that the precipitates predominantly consist of Si and Mg, corresponding to the Mg_2_Si phase that serves as the primary strengthening constituent in 6082 alloys. Figure 9d presents high-resolution transmission electron microscopy (HRTEM) images of the precipitates, while Figure 9e,f display the fast Fourier transform (FFT) images of different regions from Figure 9d. Comparative analysis with the existing literature confirms that these precipitates correspond to the β” phase commonly observed in peak-aged aluminum alloys. The absence of Al_3_Sc nanoprecipitates were detected in the Sc-Zr-6082 alloy, which could be attributed to the aging temperature (180 °C) being below the thermodynamic stability range (250–500 °C) required for Al_3_Sc phase formation [28].

According to the results in Figure 6b, the synergistic addition of Sc and Zr significantly enhances both strength and ductility in the 6082 alloy. Research in the literature [33] indicates that ductility is closely related to grain size; finer grains not only improve strength but also promote plastic deformation by increasing grain boundary density. After squeeze casting and optimized heat treatment, the grain size of the Sc-Zr-6082 alloy is significantly finer than that of the conventional 6082 alloy, with grain refinement becoming the key factor in enhancing its mechanical properties. The strengthening mechanism of grain refinement can be explained by the classical Hall–Petch relationship, as expressed by the following equation [34]:

(1) $$∆\sigma_{grain}=k(d^{-\frac{1}{2}}-{d_{0}}^{-\frac{1}{2}})$$

In the equation, parameter *k* represents the Hall–Petch slope, *d*_0_ denotes the initial grain size, and *d* corresponds to the refined grain size. The observed inverse correlation between Δ*σ*_grain_ and *d* demonstrates that decreasing grain size directly enhances strength. Elevated grain boundary density restricts dislocation slip, requiring higher stress for plastic deformation. The increased grain boundary area improves stress distribution homogeneity, thereby enhancing fracture resistance. The simultaneous ductility improvement stems from refined grains enabling more uniform strain accommodation during tensile loading. These coupled effects fundamentally explain the grain size dependence of ductility, where smaller grains generally correlate with enhanced plastic deformation capacity.

In addition, the incorporation of Sc and Zr not only promotes grain refinement but also optimizes the distribution of grain boundary precipitates. This effectively reduces brittle phases accumulation, consequently decreasing the propensity of crack initiation and propagation. This synergistic mechanism ensures simultaneous retention of ductility/fracture toughness and strength enhancement, successfully circumventing the conventional strength and plasticity trade-off.

The yield strength of aluminum alloys is governed by four principal strengthening mechanisms: precipitation hardening, grain boundary strengthening, solid solution strengthening, and dislocation strengthening [35]. The strength enhancement achieved through Sc and Zr microalloying originates from synergistic effects of multiple mechanisms, with precipitation hardening playing a dominant role through microalloying-induced modifications in precipitate morphology within the alloy matrix. The primary precipitates in age-hardening 6082 alloy include the β”, β’, and Mg_2_Si phases. Strength increases mainly due to the obstruction of dislocation motion by secondary particles [36]. When dislocations encounter secondary particles, they either pass through smaller particles or bypass larger ones, with a critical particle radius of approximately 2 nm [37]. As shown in Figure 9, the β” precipitates averaging 5 nm in size, while the β’ precipitates range from 40 to 80 nm, indicating that dislocations primarily bypass the larger precipitates. The increase in yield strength can be calculated using the Orowan equation [38].

(2) $$∆\sigma_{orowan}=0.4MGb\ln\left(2\sqrt{\frac{2}{3}}\frac{r}{b}\right)/(\pi \lambda \sqrt{1-\upsilon })$$

where *M* represents the Taylor factor, *b* is the Burgers vector, *G* is the shear modulus, and *υ* is Poisson’s ratio. The interparticle spacing *λ* is determined by the characteristics of the secondary particles, specifically their volume fraction *f* and radius *r*. The relationship between these parameters is expressed as follows [39]:

(3) $$\lambda =2\sqrt{2/3}r(\sqrt{\frac{\pi }{4f}}-1)$$

As indicated by Equations (2) and (3), high-density nanoscale precipitates, including β”, β’, and Mg_2_Si, play a crucial role in matrix strengthening. The strengthening effect intensifies with increasing precipitate density, maximizing the Orowan mechanism’s contribution. These precipitates effectively hinder dislocation motion during their migration from grain interior to boundaries, which is essential for the Sc-Zr-6082 alloy’s mechanical properties.

### 3.3. Mechanical Properties of Aluminum Alloy Components

**Figure 10:**
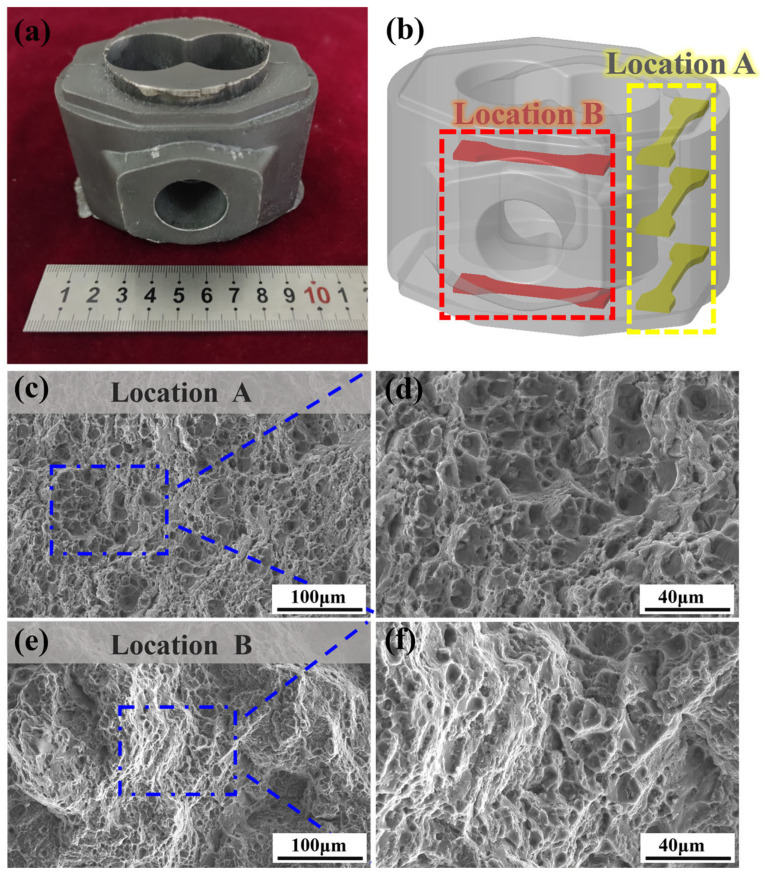
Sc-Zr-6082 alloy valve body component and the fracture morphology of tensile specimens: (**a**) Sc-Zr-6082 alloy valve body component; (**b**) schematic diagram of the selection location of tensile specimens; (**c**,**d**) fracture morphology of tensile specimens in location A; (**e**,**f**) fracture morphology of tensile specimens in location B.

The Sc-Zr-6082 alloy valve body component was manufactured via squeeze casting, with Figure 10a displaying the as-cast product. To evaluate the mechanical properties of the components, tensile tests were conducted in different regions of the component, as indicated in the schematic of testing locations shown in Figure 10b. Table 3 summarizes the mechanical properties after heat treatment from distinct locations of the component. Fracture surfaces in Figure 10c–f reveal distinct fracture mechanisms between locations. The fracture surface of location A (Figure 10c,d) exhibits numerous smooth dimples with almost no signs of brittle fracture, indicating a typical ductile fracture in this region. Location B (Figure 10e,f) displays shallow dimples, indicative of reduced ductility. This observation is consistent with the tensile test results in Table 3, indicating a clear correlation between the microstructural features and the mechanical properties.

Although the mechanical properties vary between regions due to deformation differences during the squeeze casting process, the strategic incorporation of Sc/Zr elements ensures that the overall mechanical performance of the valve body components meets product standards. This finding not only validates the effectiveness of alloy element optimization but also highlights the potential of the squeeze casting process for manufacturing aluminum alloy components with balanced strength–ductility characteristics.

## 4. Conclusions

This study systematically investigated the synergistic effects of Sc/Zr microalloying on a 6082 aluminum alloy processed by squeeze casting. Three key findings emerge:

(1) Sc and Zr co-addition induced remarkable grain refinement, achieving an equiaxed grain structure (136.36 μm average size) with 31.7% size reduction compared to the baseline alloy. The microalloying simultaneously decreased non-equilibrium eutectic phases fraction at the grain boundaries and refined coarse AlFeMnSi intermetallic phases.

(2) The Sc/Zr-modified alloy exhibited prolonged age-hardening kinetics at 180 °C, requiring 100% longer peak-aging duration (from 6 h in baseline alloy to 12 h), attributed to Sc/Zr-induced retardation of β’’-phase precipitation.

(3) The squeeze-cast Sc-Zr-6082 alloy achieved better mechanical properties with ultimate tensile strength, yield strength, and elongation reaching 358.0 MPa, 318.5 MPa, and 13.1%, respectively, representing improvements of 10.4%, 8.0%, and 71.8% compared to the 6082 alloy. This improvement was consistently demonstrated in manufactured valve bodies, where yield strengths exceeding 300 MPa and elongations above 10% were maintained across different sections, confirming the spatial homogeneity of mechanical performance.

## Figures and Tables

**Table 1:** Chemical compositions of the experimental alloys. (wt.%).

Alloy	Mg	Si	Fe	Mn	Ti	Zn	Cr	Sc	Zr	Al
6082	1.056	1.072	0.275	0.523	0.026	0.053	0.147	-	-	Bal.
Sc-Zr-6082	1.002	1.008	0.222	0.506	0.030	0.047	0.154	0.298	0.196	Bal.

**Table 2:** Tensile properties at room temperature of the experimental alloys.

Experimental Alloys	YS (MPa)	UTS (MPa)	EL (%)
6082	93 ± 1	171 ± 3	19 ± 2
Sc-Zr-6082	123 ± 5	239 ± 6	22 ± 1

**Table 3:** Mechanical properties at different locations of the Sc-Zr-6082 alloy valve body component.

Locations	YS (MPa)	UTS (MPa)	EL (%)
A	360 ± 6	372 ± 6	17 ± 1
B	329 ± 37	340 ± 20	12 ± 2

## Data Availability

The original contributions presented in this study are included in the article. Further inquiries can be directed to the corresponding author(s).
